# Family functioning and adolescent depression: A moderated mediation model of self-esteem and peer relationships

**DOI:** 10.3389/fpsyg.2022.962147

**Published:** 2022-08-09

**Authors:** Xinquan Huang, Ningning Hu, Zhengdong Yao, Biao Peng

**Affiliations:** ^1^School of Marxism, Guizhou Medical University, Guiyang, China; ^2^School of Nursing, Xinjiang Medical University, Urumqi, China; ^3^Normal College, Hunan University of Arts and Science, Changde, China

**Keywords:** adolescent, family functioning, self-esteem, peer relationships, depression

## Abstract

In consideration of family system theory, the vulnerability model of depression, and the stress buffering model of social support, the current study examined the effect of family functioning on adolescent depression, the mediating effect of self-esteem, and the moderating effect of peer relationships. A sample of Chinese adolescents (*n* = 562, 47.15% male, 52.85% female, mean age 14.33 years, SD = 1.81 years) completed questionnaires regarding family functioning, depression, self-esteem, and peer relationships. The results showed that: (1) family functioning had a significant negative predictive effect on adolescent depression; (2) self-esteem plays a mediating role between family functioning and adolescent depression; and (3) peer relationships have a moderating effect on the relationship between self-esteem and adolescent depression, supporting the moderated mediation model. These results reveal the influence mechanism of family functioning on adolescent depression and have implications for adolescent depression intervention.

## Introduction

Depression is a common mental disorder characterized by sadness, inability to experience happiness, self-criticism, and physical symptoms such as poor concentration, fatigue, loss of energy, and disturbed sleep or appetite (World Health Organization, 2017). Depression can seriously affect adolescents’ physical and mental development (Li and Li, 2022), leading to truancy and school avoidance, delinquency, and confrontation, and can increase their risk of substance abuse and, in severe cases, even suicide (Egger et al., 2003; Rowe et al., 2006; Johnson et al., 2018). A recent meta-analysis showed that the overall detection rate of depressive symptoms among Chinese adolescents was 28.4% (Liu et al., 2020), which indicates that more attention must be paid to the issues of depression among Chinese adolescents.

Previous studies have found that poor family functioning can make individuals depressed (Cheng et al., 2022), and family functioning plays an important role in the development, process, and relapse of depression (Keitner et al., 1995; Sireli and Soykan, 2016). However, few studies have explored the mediating and moderating mechanisms of family functioning on depression. According to the ecological systems theory, family, peer, and individual psychological characteristics (e.g., self-esteem, etc.) have a strong impact on an individual’s mental health (Bronfenbrenner, 1989). The roles of self-esteem and peer relationships in the influence of family functioning on adolescent depression deserve attention. Therefore, the current study aimed to determine the association between family functioning and adolescent depression (a psychological disease and negative emotion) and to explore the mediating role of self-esteem and the moderating role of peer relationships in Chinese adolescents.

### Family functioning and adolescent depression

Family functioning is the function of the family system itself, which refers to the ability of the family to function effectively to meet basic needs and manage conflicts (Jona et al., 2017). The circumplex model of marital and family systems considers family as functioning in three dimensions: family cohesion, flexibility (initially called adaptability), and communication (Olson, 2000). Family cohesion is the ability to maintain strong emotional bonds among family members. Flexibility focuses on how the family system balances stability and change. Good communication promotes family cohesion and flexibility (Olson et al., 2019).

Numerous theoretical and empirical studies have linked family dysfunction to depression. The effect of family functioning on adolescent depression can be explained using the family system theory. According to family system theory, the better the overall function of the family system, the better the psychological state and behavioral performance of its members, leading to less depression or other emotional and behavioral problems (Beavers and Hampson, 2000). Family cohesion can provide a warm family environment and positive emotional support, thus reducing the likelihood of adolescents developing depression or other forms of adverse emotional distress. Family flexibility, on the other hand, enables families to cope with change and reduces the impact of negative events on adolescents’ mental health (Nam et al., 2016). Positive communication leads to less family conflict, enhancing family adaptability and cohesion and thus playing a protective role in adolescent mental health (Yee and Sulaiman, 2017). Empirical studies have found that family functioning has a significant influence on adolescent depression (Lin et al., 2008; Sireli and Soykan, 2016; Shao et al., 2020), that family cohesion is significantly negatively correlated with depression (Kashani et al., 1995; Roley et al., 2014; Zahra and Saleem, 2020), and that low family adaptability and poor family communication play important roles in adolescent depression (Gladstone et al., 2005; Sheeber et al., 2007; Lee et al., 2017). A recent meta-analysis showed that family dysfunction is closely related to depressive symptoms, and that family functioning is an important predictor of individual depression (Guerrero-Muñoz et al., 2021). Based on the above-mentioned literature, then, we proposed our first hypothesis as follows:

*Hypothesis 1*: There is a significantly negative correlation between family functioning and adolescent depression.

### The mediating role of self-esteem

Adolescent depression is not only affected by family functioning, but also related to individual characteristics. Self-esteem, as an individual characteristic, has been shown to have a particularly important influence on depression. Self-esteem is a set of thoughts and feelings regarding one’s own worth and importance (Rosenberg, 1965). The vulnerability model of depression suggests that low self-esteem is a predisposition factor for depression, and that low self-esteem leads to depression through both interpersonal (e.g., seeking excessive reassurance, seeking negative feedback, social avoidance) and intrapersonal pathways (e.g., ruminating about negative aspects of the self; Orth et al., 2008). A large number of studies have confirmed the relationship between self-esteem and depression, also noting that implicit self-esteem predicts future depressive symptoms (Franck et al., 2007), that low self-esteem predicts adult depression (Steiger et al., 2014), and that low self-esteem not only directly predicts depression, but also has an impact on depression through psychological inflexibility (Peng et al., 2021b).

The formation and development of adolescent self-esteem has been shown to be the result of the interactions between social, family, and school cultures, of which family is regarded as the most important setting for the building up of self-esteem (Bradshaw, 1996). According to the circumplex model of marital and family systems, family cohesion can foster self-esteem by promoting family members’ emotions and creating a warm family atmosphere, while flexibility can provide a good environment for the development of self-esteem by maintaining family stability and balance (Olson et al., 2019). Studies have shown that emotional communication, positive communication, problem solving and family rules are significantly positively correlated with self-esteem (Liu and Zhao, 2018), while family cohesion and adaptability are significantly positively correlated with self-esteem (Kawash and Kozeluk, 1990; Merkaš and Brajša-Žganec, 2011; Mao et al., 2015). Poor family functioning reduces adolescents’ self-esteem (Yen et al., 2013) and increases depressive symptoms (Guerrero-Muñoz et al., 2021). Good family functioning can promote adolescent self-esteem (Shi et al., 2017), reducing depressive symptoms (Lorenzo-Blanco et al., 2016). Self-esteem plays a mediating role in family functioning and mental health (Man et al., 2017; KavehFarsani et al., 2020). Based on these aforementioned findings, then, we proposed our second hypothesis as follows:

*Hypothesis 2*: Self-esteem plays a mediating role between family functioning and adolescent depression.

### The moderating role of the peer relationship

It is well-known that low self-esteem predicts adolescent depression (Orth et al., 2008), but not all adolescents with low self-esteem exhibit the same levels of depression. One possible reason for this phenomenon is differences in adolescents’ peer relationships. In terms of the psychological definition of stress, low self-esteem is closely related to high stress assessment (Cohen and Wills, 1985). According to the stress buffering model of social support, positive peer relationships, as an important social support force, can effectively relieve pressure experienced by adolescents due to low self-esteem and thereby play a positive role in promoting adolescent mental health (Sarason et al., 1990; Rueger et al., 2016). In other words, peer relationships may act as moderators to mitigate the negative effects of low self-esteem.

A peer relationship is a kind of parallel and equal interpersonal relationship established and developed through the process of communication between peers or individuals at the same level of psychological development (Zhou et al., 2015). With the rapid development of adolescent physiology and psychology, adolescent peer relationships play an increasingly important role in the adolescent social support system. Studies have shown that positive peer relationships can provide adolescents with information, as well as emotional and value support (Xu and Zhang, 2011), all of which can enhance positive behavioral traits (Schwartz et al., 2010) and protect adolescents from experiencing victimization and depression (Abou-ezzeddine et al., 2007; Healy and Sanders, 2018). This can effectively moderate the relationship between adolescent life stresses and depression as well as against other negative affects (Rubin et al., 1992; Deng et al., 2021). This can also moderate the relationship between adolescent empathy and depression (Yan and Li, 2021) and the relationship between adolescent self-esteem and pathological Internet use (Zhang et al., 2013). At the same time, high quality peer relationships can also affect core self-evaluation (Dong et al., 2022), promote the formation of one’s positive self-concept thus forming a more mature level of cognition, enhancing emotional regulation ability (Wang, 2006), reducing social withdrawal behavior, and abating the association between negative self-cognition and adolescent depression (Tang et al., 2018; Dong et al., 2022). Therefore, high quality peer relationships may alleviate the impact of low self-esteem on adolescent depression, while low quality peer relationships may increase the impact of low self-esteem on adolescent depression. Furthermore, peer relationships may play a moderating role in the relationship between adolescent self-esteem and depression. Based on these understandings, then, we proposed the third hypothesis as follows:

*Hypothesis 3*: Peer relationships play a moderating role between self-esteem and adolescent depression.

### The current study

**Figure 1:**
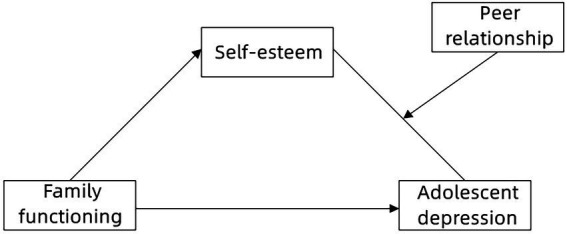
The proposed moderated mediation model.

Combining ecological systems theory, family system theory, and social support theory, the present study aimed to explore the complex moderated mediation model underlying the association between family functioning and adolescent depression (Figure 1). The purposes of our study were to test (a) whether family functioning could significantly predict adolescent depression; (b) whether self-esteem would mediate the association between family functioning and adolescent depression; and (c) whether peer relationships could moderate the association between self-esteem and adolescent depression.

## Materials and methods

### Participants

The participants in this study were recruited from two middle schools in Hunan and Guizhou provinces, China. A total of 700 questionnaires were sent out, and 562 valid questionnaires were collected after excluding invalid ones such as regular answers and missed answers. Participants were aged 12–19 years old (*M* = 14.33, *SD* = 1.81), 265 (47.15%) were boys and 297 (52.85%) were girls. In terms of their studies, 139 (24.73%) were in 7th grade, 138 (24.56%) were in 8th grade, 107 (19.04%) were in 9th grade, 66 (11.74%) were in 10th grade, 64 (11.39%) were in 11th grade, and 48 (8.54%) were in 12th grade.

### Procedure

The study was approved by the Ethical Review Committee of Guizhou Medical University, as well as by the investigated schools and local education departments. Before data collection, participants were informed of the purpose, confidentiality, and anonymity of this study, and informed consent was obtained. Participants completed the self-report questionnaires in class under the guidance of trained researchers.

### Measurements

#### Family APGAR index

The Family APGAR Index (APGAR) was compiled by Smilkstein (1978) to measure family functioning. It has five items which represent adaptability, partnership, growth, affection, and resolve. Each item of the APGAR is rated using a three-point Likert scale that ranges from 0 (hardly ever) to 2 (almost always). The Chinese version of the APGAR has good reliability and validity among Chinese teenagers (Yen et al., 2013; Wang et al., 2019). In this study, the Cronbach α coefficient of the APGAR was 0.92. A score of 0–3 is classified as severe family dysfunction, 4–6 as moderate family dysfunction, and 7–10 as good family dysfunction (i.e., a low level of family dysfunction).

#### The Rosenberg self-esteem scale

The Rosenberg Self-Esteem Scale (RSES) was developed by Rosenberg (1965) to measure self-esteem. It is structured as a single dimension and contains ten items. Each item of the RSES is rated using a four-point Likert scale that ranges from 1 (very nonconforming) to 4 (very conforming). The revised Chinese version of the Rosenberg Self-esteem Scale (RSES-R) has been shown to have good reliability and validity in Chinese adolescents (Wei et al., 2018). In this study, the Cronbach α coefficient of the RSES-R was 0.89.

#### Youth self-rating index

The youth self-rating index (YSR) was developed by Achenbach (1991). The current study used only the depression subscale of the YSR, which comprises 16 items which are rated using a three-point Likert scale ranging from 1 (disagree) to 3 (Quite agree with). The Chinese version of the YSR has good reliability and validity in Chinese adolescents (Liu et al., 1997). In this study, the Cronbach α coefficient of the YSR depression subscale was 0.94.

#### The peer relationship scale

The peer relationship scale was compiled by Asher et al. (1984) and revised by Zhang (2008) to be translated into Chinese. It measures three dimensions—welcome, exclusion, and loneliness—using a total of 16 items. Each is scored by up to 4 points, and 10 of the items are scored in reverse. After the reverse scoring conversion, the total score of the scale is calculated. The higher the total score, the better the peer relationship. The Cronbach *α* coefficient of the scale in this study was 0.93.

#### Data analysis strategy

Statistical analyses were performed using SPSS 24.0 and Hayes’ (2013) PROCESS macro. First, descriptive statistics and correlation analysis were performed on the data using SPSS 24.0. Next, we used Hayes’ (2013) PROCESS macro (Model 4) to examine the mediating effect of self-esteem on the relationship between family functioning and adolescent depression. We then used Hayes’ (2013) PROCESS macro (Model 14) to examine whether the peer relationship moderated this mediation process. Finally, the simple slope test was used to explain the moderation effect. All variables were standardized before mediating and moderating effects were examined.

## Results

### Preliminary analysis

Harman’s single-factor test was used to test the effect of common method bias. Exploratory factor analysis was conducted on the four variables together. The results showed that there were six factors with characteristic roots greater than 1, and the explanation rate of the first factor was 35.41%—less than 40%—indicating that there was no common method bias in this study. Common method bias was further tested by confirmatory factor analysis (CFA). The results showed that the single-factor CFA model did not meet the standard of good fitting (*χ*^2^/*df* = 6.98, CFI = 0.65, TLI = 0.63, RMSEA = 0.103). Therefore common method bias is not serious (Iverson and Maguire, 2000).

The mean, standard deviation, and Pearson correlation of all variables are shown in Table 1. The teacher–student relationship was negatively associated with deviant peer affiliation and bullying perpetration. Deviant peer affiliation and peer pressure were positively associated with bullying perpetration. Additionally, peer pressure was positively associated with deviant peer affiliation.

**Table 1:** Descriptive statistics and correlations for all variables.

	Family functioning	Peer relationship	Self-esteem	Depression
Family functioning	1			
Peer relationship	0.49^**^	1		
Self-esteem	0.54^**^	0.70^**^	1	
Depression	−0.46^**^	−0.64^**^	−0.67^**^	1
*M*	6.08	49.87	28.43	24.59
SD	2.53	9.61	5.27	6.64

**P < 0.01.

### Testing for mediation effect

**Figure 2:**
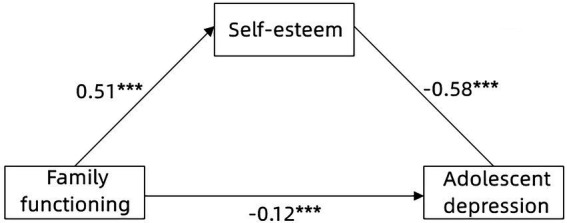
The mediating effect of self-esteem on the relationship between family functioning and adolescent depression. ***P < 0.001.

Hypothesis 2 proposed that self-esteem plays a mediating role between family functioning and adolescent depression. To verify this hypothesis, the PROCESS macro (Model 4) was used to test the mediating effect (see Figure 2). As shown in Table 2, after controlling for gender and grade, family functioning significantly positively predicted self-esteem (*β* = −0.51, *p* < 0.001), and self-esteem significantly negatively predicted adolescent depression (*β* = −0.58, *p* < 0.001). The direct effect of family functioning on adolescent depression was significant (*β* = −0.12, *p* < 0.001). The bias-corrected percentile bootstrap method showed that family functioning had a significant indirect effect on adolescent depression through self-esteem: *ab* = 0.30, SE = 0.04, 95% CI = [−0.37, −0.23]. The mediating effect accounted for 71.43% of the total effect. Therefore, Hypothesis 2 is supported.

**Table 2:** Testing the mediation effect of family functioning on adolescent depression.

	Model 1 Adolescent depression		Model 2 Self-esteem		Model 3 Adolescent depression	
	*β*	*t*	*β*	*t*	*β*	*t*
Gender	0.11	3.06^**^	−0.05	−1.37	0.09	2.76^**^
Grade	0.13	3.27^**^	−0.08	−2.26^*^	0.08	2.40^*^
Family functioning	−0.42	11.02^***^	0.51	13.90^***^	−0.12	−3.35^***^
Self-esteem					−0.58	−16.02^***^
*R^2^*	0.24		0.30		0.48	
*F*	59.12^***^		78.40^***^		128.78^***^	

*P < 0.05, **P < 0.01 and ***P < 0.001.

### Testing for moderated mediation

Hypothesis 3 proposes that peer relationships play a moderating role in the relationship between self-esteem and adolescent depression. To verify this hypothesis, the PROCESS macro (Model 14) was used to test the moderating effect. As shown in Table 3, peer relationships negatively predicted adolescent depression (*β* = −0.26, *p* < 0.001). Peer relationships and self-esteem show significant interaction with adolescent depression (*β* = −0.15, *p* < 0.001). The bias-corrected percentile bootstrap method showed that peer relationships moderated the indirect effect of family functioning on adolescent depression, and the moderated mediation index was –0.19, SE = 0.03, 95%CI = [−0.26, −0.14]. When the peer relationship level was low (i.e., one SD below the mean), self-esteem significantly mediated the relationship between family functioning and adolescent depression, and the mediating index was *ab* = −0.27, 95%CI = [−0.35, −0.21]. At the same time, when the peer relationship level was high (i.e., one SD above the mean), self-esteem had a significant mediating effect on family functioning and adolescent depression, and the mediating index was *ab* = −0.11, 95%CI = [−0.17, −0.06].

**Table 3:** Testing the moderated mediation effect of peer relationships on self-esteem and adolescent depression.

	Model 1 Self-esteem		Model 2 Adolescent depression	
	*β*	*t*	*β*	*t*
Gender	−0.05	−1.37	0.08	2.85^**^
Grade	−0.08	−2.26^*^	0.07	2.52^*^
Family functioning	0.51	13.90^***^	−0.08	2.32^*^
Self-esteem			−0.38	−9.01^***^
Peer relationships			−0.26	−6.48^***^
Self-esteem × peer relationship			0.15	6.57^***^
*R^2^*	0.30		0.56	
*F*	78.40^***^		118.40^***^	

*P < 0.05, **P < 0.01 and ***P < 0.001.

**Figure 3:**
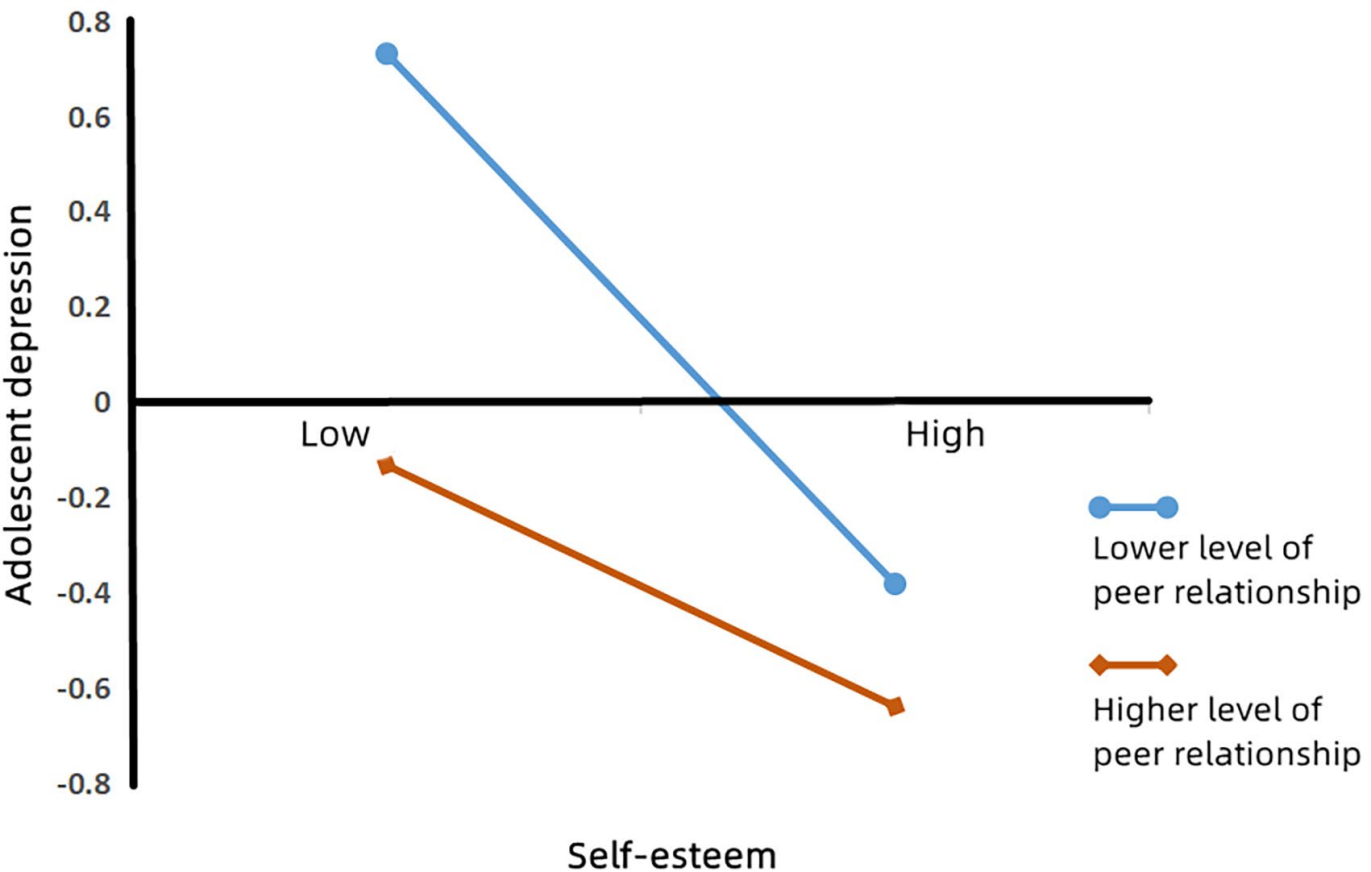
Model of test for simple slopes showing moderating influence of peer relationships on association between family functioning and adolescent depression.

To understand the essence of the moderating effect, a simple slope test was conducted (Aiken and West, 1991). As shown in Figure 3, self-esteem had a significant negative predictive effect on adolescent depression when peer relationships were low (i.e., one SD below the mean; *β*_simple_ = −0.56, *p* < 0.001). When the level of peer relationships was high (i.e., one SD above the mean), the negative predictive effect of self-esteem on adolescent depression decreased (*β*_simple_ = −0.25, *p* < 0.001). Therefore, high quality peer relationships can alleviate the impact of low self-esteem on adolescent depression; low quality peer relationships can increase the impact of low self-esteem on adolescent depression; and peer relationships play a moderating role in the relationship between self-esteem and adolescent depression.

## Discussion

Although family system theory and a large number of empirical studies support that family functioning plays an important role in the development, process, and relapse of adolescent depression (Keitner et al., 1995; Beavers and Hampson, 2000; Sireli and Soykan, 2016), there are few studies into the mediating and moderating mechanisms of family functioning on adolescent depression.

Based on ecological systems theory among others, this study explored the mediating role of self-esteem and the moderating role of peer relationships in the relationship between family functioning and adolescent depression.

### The relationship between family functioning and adolescent depression

The current study found a significant negative correlation between family functioning and adolescent depression, which is consistent with Hypothesis 1. This result can be explained by the circumplex model of marital and family systems (Olson, 2000). Good family functioning can have a protective effect on adolescent mental health and reduce the risk of depression. High family cohesion can provide a warmer family environment and positive emotional support for adolescents, leading them to form good parent–child relationships and ultimately reducing the risk of depression (Edwards and Clarke, 2004). High family adaptability (or flexibility) can help adolescents effectively cope with the impacts of external negative life events and reduce their risk of depression (Nam et al., 2016). Good family communication can enhance family cohesion, adaptability, and help maintain the stability and balance of the family system, thus reducing the risk of depression in adolescents (Yee and Sulaiman, 2017). Conversely, poor family functioning increases the risk of depression in adolescents.

### The mediating role of self-esteem

This study also found that self-esteem plays a mediating role in the relationship between family functioning and adolescent depression, confirming our Hypothesis 2. Self-esteem is a series of thoughts and feelings about an individual’s own worth and importance (Rosenberg, 1965). As an evaluation and emotional dimension of self-concept, self-esteem is considered to be equal to self-respect, self-evaluation, and self-worth (Baumeister, 1997; Harter, 1999). Individuals with high self-esteem have more positive self-evaluation and self-experience, while those with low self-esteem experience more self-rejection, self-dissatisfaction, and self-contempt.

Family support, parental emotional warmth, and a positive parent–child relationship are all important factors influencing a child’s self-esteem (Franco and Levitt, 1998; Bulanda and Majumdar, 2009; Peng et al., 2021a). According to the circumplex model of marital and family systems, family functions manifest primarily as family cohesion, adaptability (or flexibility), and communication, which can affect both adolescent depression and self-esteem. High family cohesion can result in children feeling loved, accepted, and supported, helping them form a good parent–child relationship, and lead to children themselves forming a more positive sense of self-evaluation. However, low family cohesion will result in children feeling rejection and neglect, forming bad parent–child relationships, and then causing the children form a negative sense of self-evaluation. High family adaptability helps family members understand their roles, functions, and power, as well as family expectations, and thus help children respond effectively to life events. By coping with events effectively and increasing their ability to control their environment, children become confident and develop a more positive sense of self-esteem (Rezaei-Dehaghani et al., 2015). Positive communication promotes positive self-esteem by reducing family conflict, enhancing family cohesion, and increasing adaptability.

Low self-esteem, however, is an significant risk factor for depression in adolescents. According to the vulnerability model of depression, individuals with low self-esteem display behavioral characteristics such as seeking excessive comfort or negative feedback and actively being socially avoidant, as well as internal characteristics such as ruminating about negative aspects of one’s self which can then lead to depression (Orth et al., 2008). In addition, according to acceptance and commitment therapy, people with low self-esteem tended to show more cognitive fusion and experiential avoidance, both of which are also important causes of depression (Peng et al., 2021b).

### The moderating role of peer relationships

This study found that peer relationships mediated the second stage of the indirect relationship between family functioning and adolescent depression through self-esteem, confirming our Hypothesis 3. Among adolescents with a low level of peer relationships, lower self-esteem was significantly associated with higher depressive symptoms. However, this relationship became weaker in adolescents with a high level of peer relationships. In other words, a high level of peer relationships can mitigate the effect of low self-esteem on adolescent depression.

This result is consistent with the stress buffering model of social support (Sarason et al., 1990; Rueger et al., 2016). When faced with life events, adolescents with low self-esteem are more likely to underestimate their ability to cope, which leads to greater stress and ultimately mental health problems such as depression. A high level of peer relationships can provide emotional support for those with low self-esteem, helping them cope with the stress brought on by negative self-evaluation, promoting the formation of a more positive self-concept and thereby alleviating the impact of low self-esteem on adolescent depression.

This result is consistent with the risk-enhancing hypothesis (Luthar et al., 2015) which assumes that one risk factor (e.g., low peer relationships) can exacerbate the negative effects of another risk factor (e.g., low self-esteem). Low peer relationships causes adolescents with low self-esteem to lack effective communication and support, making them more likely to believe in thoughts of negative self-evaluation and thus increasing the impact of low self-esteem on depression.

### Limitations and practical implications

Although this study has begun to uncover the mediating and moderating mechanisms at work between family functioning and adolescent depression, there are still some limitations to the current study that need to be considered. First, this study is a cross-sectional design, which cannot reveal the change of variables over time. Future studies should consider using a cross-lagged panel model to analyze the mediating effect of longitudinal data. Second, the data in this study are collected by a self-report method which may have been affected by the social expectation effect. At the same time, the potential method variances were not controlled or examined. Future studies should consider using multi-channel collection (e.g., teacher or parent reports) to improve research validity. Third, the samples of this study are from only two middle schools from two provinces in China, so the results of this study cannot be extended to adolescents from other cultural backgrounds. Therefore, it is necessary to expand the sample in the future, and in particular to conduct research on adolescents from different cultural backgrounds.

Despite these limitations, however, the current study nonetheless has theoretical and practical significance. Theoretically, this study aimed to explore the moderated mediation mechanism between family functioning and adolescent depression, and expands the existing understandings of adolescent depression. It also improves our understandings of the mechanism at play in the relationship between family functioning and adolescent depression. From a practical point of view, this study provides guidance for adolescent depression prevention or intervention. First, consistent with previous findings, this study suggests that positive family functioning is an important protective factor for adolescent depression. Therefore, parents should enhance family cohesion, adaptability, and positive communication to ensure family functioning is as normal and effective as possible, so as to effectively limit the chances of depression in adolescents. Second, parents and schools should cultivate adolescents’ positive self-esteem so that adolescents can effectively prevent depression through positive self-esteem. Third, considering the moderating role of peer relationships on the relationship between self-esteem and adolescent depression, schools should pay attention to the cultivation of multiple levels of positive peer relationships among adolescents to ensure that adolescents receive adequate peer support, so as to alleviate the impact of low self-esteem on adolescent depression.

## Data availability statement

The data analyzed in this study is subject to the following licenses/restrictions: The datasets generated for this study are available upon request to the corresponding authors. Requests to access these datasets should be directed to pengbiao220@qq.com.

## Ethics statement

The studies involving human participants were reviewed and approved by Guizhou Medical University. Written informed consent to participate in this study was provided by the participants’ legal guardian/next of kin.

## Author contributions

XH, ZY, and BP designed the study protocol. XH drafted the manuscript. BP performed the statistical analysis and guided the first draft of the paper. ZY guided the interpretation of the results and edited the final manuscript. NH completed the literature review and participated in the study design and interpretation analysis. All authors contributed to the article and approved the submitted version.

## Conflict of interest

The authors declare that the research was conducted in the absence of any commercial or financial relationships that could be construed as a potential conflict of interest.

## Publisher’s note

All claims expressed in this article are solely those of the authors and do not necessarily represent those of their affiliated organizations, or those of the publisher, the editors and the reviewers. Any product that may be evaluated in this article, or claim that may be made by its manufacturer, is not guaranteed or endorsed by the publisher.
